# Antiplatelet therapy is not associated with increased risk of complications after lumbar puncture

**DOI:** 10.1007/s00415-024-12864-6

**Published:** 2024-12-24

**Authors:** Laura Stichaller, Nik Krajnc, Fritz Leutmezer, Elisabeth Stögmann, Friedrich Zimprich, Tobias Zrzavy, Thomas Berger, Gabriel Bsteh

**Affiliations:** 1 Department of Neurology, Medical University of Vienna, Waehringer Guertel 18-20, 1090 Vienna, Austria; 2 Comprehensive Center for Clinical Neurosciences and Mental Health, Medical University of Vienna, Vienna, Austria

**Keywords:** Lumbar puncture, Complication, Risk factors, Post-dural puncture headache, Antiplatelet therapy

## Abstract

**Background:**

Lumbar puncture (LP) is a critical diagnostic procedure in the evaluation of neurological diseases. Although considered safe, complications such as post-dural puncture headache (PDPH), back pain, subdural hematoma or venous sinus thrombosis may still occur. Whether the use of antiplatelet therapy (APT) increases the risk of complications after LP, remains unclear.

**Methods:**

This retrospective observational study included 783 patients who underwent diagnostic LP. We employed multivariate logistic regression models with complications as the dependent variable, and APT as the independent variable, adjusting for potential confounders.

**Results:**

Among 783 patients included (54.0% female, median age 48 years [IQR 33–64], median BMI 24.7 kg/m^2^ [IQR 21.8–28.3], 111 [14.2%] receiving APT), complications were observed in 182 (23.2%) patients. The most common complications were PDPH and back pain in 152 (19.4%) and 42 (5.4%) patients, respectively. Venous sinus thrombosis occurred in one (0.1%) patient. In the multivariate logistic regression model, younger age (OR 1.49 per 10 years, 95% CI 1.32–1.69, *p* < 0.001) and female sex (OR 1.74, 95% CI 1.19–2.54, *p* = 0.005) were associated with higher likelihood of complications, whereas APT (OR 0.63, 95% CI 0.30–1.36, *p* = 0.241) and the final diagnosis were not.

**Conclusion:**

Complications following LP occur in approximately one fourth of patients, with younger age and female sex being significant risk factors. As APT is not associated with increased risk of complications, withholding LP in patients on APT may not be necessary.

**Supplementary Information:**

The online version contains supplementary material available at 10.1007/s00415-024-12864-6.

## Introduction

Lumbar puncture (LP) is one of the most important diagnostic procedures in neurology, providing insight into the pathology of the central nervous system (CNS) [1, 2]. It is most commonly performed for suspected infectious, autoimmune, degenerative, and vascular diseases of the CNS [3]. Although performed routinely and considered safe, it is associated with a non-negligible risk of complications, of which post-dural puncture headache (PDPH) appears to be the most common [4–6]. It occurs in up to 50% of patients, most commonly in young women, and is also associated with the use of traumatic needles during the procedure [4, 5, 7, 8]. Although the exact pathophysiological mechanism behind PDPH is unclear, it is thought to be related to the leakage of cerebrospinal fluid (CSF) through the dural hole created by the needle, resulting in low CSF pressure [9, 10]. However, not all patients with PDPH have low CSF pressure, and not all patients with a significant leak develop orthostatic headache—the rate of complications also depend on several other factors, including headache history, anxiety about the procedure and the experience of the physician [5, 7, 11]. Back pain following LP is reported in approximately 17% of patients and usually resolves within 48 h [7, 12]. Although PDPH and back pain are generally harmless and self-limiting, LP can also be associated with rare yet serious complications, including subdural hematoma, infection and venous sinus thrombosis [13–18]. While LP is contraindicated in patients with coagulopathy and those on oral anticoagulants due to the increased risk of bleeding, very little is known about the association between the complication rate and antiplatelet therapy (APT) [19–24].

The aim of this study was to assess the incidence of complications following LP and their potential risk factors in a real-world cohort, with a particular focus on the role of APT.

## Methods

### Patients and definitions

**Fig. 1:**
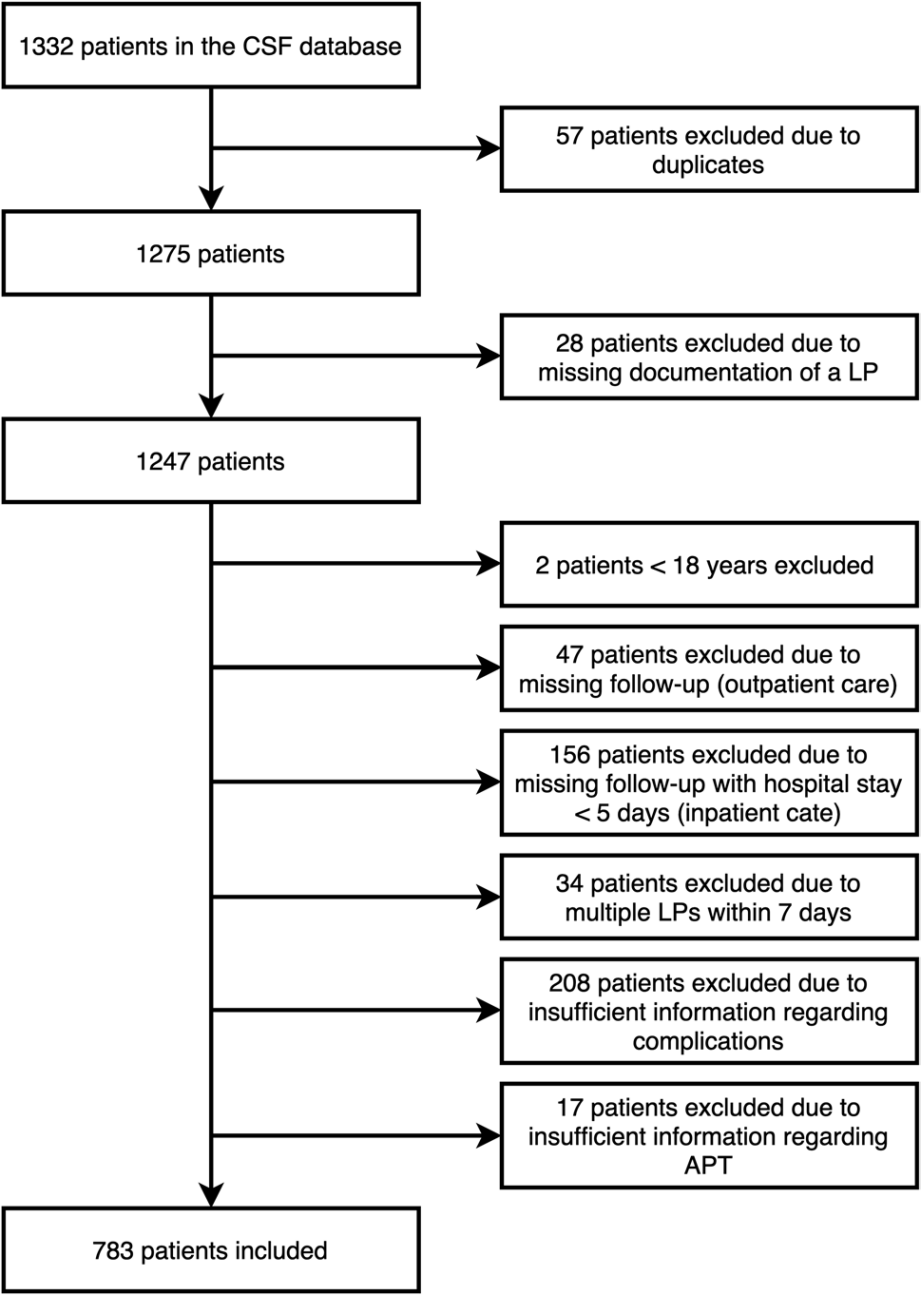
Flowchart of patients’ selection based on the inclusion and exclusion criteria

For this retrospective observational study, patients who received at least one LP at the Department of Neurology, Medical University of Vienna between January 1st, 2012 and December 31st, 2021, and whose cerebrospinal fluid (CSF) sample has been preserved in the local CSF database were screened. Contraindications for lumbar puncture, such as increased intracranial pressure, infection at the puncture site, and coagulopathy, were carefully evaluated and excluded through a thorough clinical assessment, appropriate imaging technique, and laboratory tests for coagulation status like thrombocytopenia. Patients who underwent more than one LP and had multiple CSF samples available were only included once, i.e., at the time of the chronologically first sample asservation. Patients who had no sufficient clinical follow-up to reliably assess complications after LP, and in whom a further LP was performed within 7 days were excluded. The selection process based on the inclusion and exclusion criteria is shown in Fig. 1.

All documented complications occurring during the first 4 weeks after LP were recorded. The primary outcome was the occurrence of any complication after LP. Secondary outcomes included the occurrence of PDPH, back pain, local bleeding requiring a compression bandage, local infection at the puncture site, systemic infection, subdural hematoma, and venous sinus thrombosis. PDPH was defined according to the current ICHD-3 criteria as any headache that occurs within 5 days after LP [25]. Back pain was defined as puncture-related if it occurred in the first 5 days after LP, whereas other secondary outcomes could occur within four weeks. Patients who were admitted to the hospital for LP for at least 5 days and had no available clinical follow-up were considered to have no complications. The parameters collected included demographic data, patient-related parameters such as APT-status, indication of LP, CSF parameters, procedural parameters and diagnosis after LP (Supplemental Table 1). The diagnoses after LP were categorized into neurological and non-neurological, with neurological diagnoses being further classified as either acute or chronic inflammatory and non-inflammatory. Non-neurological diagnoses, designated as diagnoses of exclusion, were characterized by symptoms not attributable to pathology of the peripheral or central nervous system and were corroborated by unremarkable paraclinical findings, including normal CSF analysis.

#### Standard protocol approvals, registrations, patient consents, and reporting

The study was approved by the ethics committee of the Medical University Vienna (ethical approval number: 1290/2022). Since this was a retrospective study, the requirement for written informed consent from study participants was waived by the ethics committee. This study adheres to the reporting guidelines outlined within the Strengthening the Reporting of Observational Studies in Epidemiology (STROBE) Statement.

### Statistics

Statistical analysis was performed using SPSS 26.0 (SPSS Inc, Chicago, IL, USA). Categorical variables were expressed in frequencies and percentages, continuous variables as mean and standard deviation (SD) or median and interquartile range (IQR) as appropriate. Continuous variables were tested for normal distribution by the Kolmogorov–Smirnov test with Lilliefors correction.

The incidence of complications was compared univariately using the chi-square test. Multivariate logistic regression models were calculated with the occurrence of any complication (primary outcome) as the dependent variable and APT as the independent variable, adjusted for sex, age, BMI, and the final diagnosis (acute inflammatory neurological, chronic inflammatory neurological, non-inflammatory neurological and non-neurological). We conducted sensitivity analyses for impact of other APT than acetylsalicylic acid and dual APT by leave-one-out design.

All predefined confounders as well as variables with a univariate association with the primary outcome below the significance level of *p* < 0.2 were included as covariables. The analyses of secondary outcomes were performed in the same way.

The robustness of all regression models to unidentified confounding factors (bias) was quantified using the Rosenbaum sensitivity test according to Hodges-Lehmann Gamma [26].

Regression models were checked for collinearity by variance inflation factor (VIF) excluding all variables if the VIF was > 2.0, corresponding to an *R*^2^ of 0.50. Missing values were handled by multiple (20 times) imputation using the missing not at random (MNAR) approach with pooling of estimates according to Rubin’s rules [27]. Multiple imputation was only used if no less than 5% and no more than 40% of the values were missing. The significance level was set at a two-sided *p* value < 0.05.

## Results

Overall, 783 patients were included in the study, of which 111 patients received antiplatelet treatment. (Supplemental Table 2). Characteristics of the study cohort are shown in Table 1. Complications were documented in 182 (23.2%) patients.

**Table 1:** Demographics of the study cohort

	Study cohort (*n* = 783)
Female^a^	423 (54.0)
Age (years)^b^	48 (33–64)
BMI (kg/m^2^)^b^	24.7 (21.8–28.3)
APT^a^	111 (14.2)
Acetylsalicylic acid 100mg^a^	95 (12.1)
Clopidogrel 75mg^a^	11 (1.4)
Dual APT^a^	5 (0.6)
Complications^a^	182 (23.2)
PDPH^a^	152 (19.4)
Back pain^a^	42 (5.4)
Venous sinus thrombosis^a^	1 (0.1)
Diagnosis	
Neurological^a^	727 (92.8)
Acute inflammatory^a^	101 (12.9)
Chronic inflammatory^a^	245 (31.3)
Non-inflammatory^a^	381 (48.7)
IIH^a^	26 (3.3)
Non-neurological^a^	56 (7.2)
Setting	
Acute^a^	11 (1.4)
Elective^a^	772 (98.6)
Modality	
Bedside^a^	780 (99.6)
CT-guided^a^	3 (0.4)
Comorbidities	
Cardiovascular^a^	182 (23.2)
Metabolic^a^	105 (13.4)
Renal^a^	24 (3.1)
Gastrointestinal^a^	49 (6.3)
Hematological^a^	15 (1.9)

*APT* antiplatelet therapy, *BMI* body mass index, *CT* computed tomography, *IIH* idiopathic intracranial hypertension, *PDPH* post-dural puncture headache

^a^Number (%)

^b^Median (IQR)

### Primary outcome

**Fig. 2:**
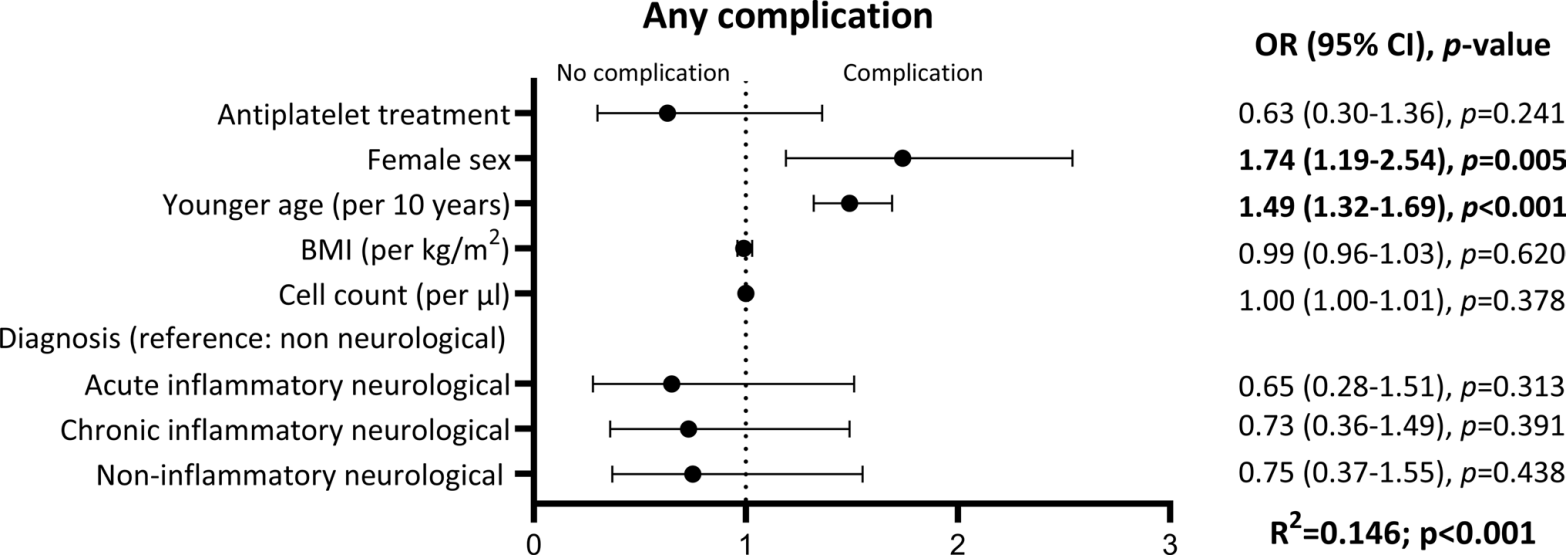
Multivariate logistic regression model for the primary outcome (occurrence of any complication). *APT* antiplatelet therapy, *BMI* body mass index

Complications after LP occurred in 11 (9.9%) and 171 (25.4%) patients with and without APT, respectively (*p* < 0.001). Univariately, APT was associated with lower likelihood of complications after LP (OR 0.32; 95% CI 0.17–0.62; *p* < 0.001) (Supplemental Table 3). In the multivariate logistic regression model, younger age (OR 1.49 per 10 years; 95% CI 1.32–1.69; *p* < 0.001) and female sex (OR 1.74; 95% CI 1.19–2.54; *p* = 0.005) were associated with higher likelihood of complications, whereas APT (OR 0.63, 95% CI 0.30–1.36, *p* = 0.241) and other covariates (final diagnosis, BMI and CSF parameters) were not (Fig. 2).

Prespecified sensitivity analyses excluding patients with other APT than acetylsalicylic acid and dual APT did not significantly alter the overall results or impact individual variables.

### Secondary outcomes

**Fig. 3:**
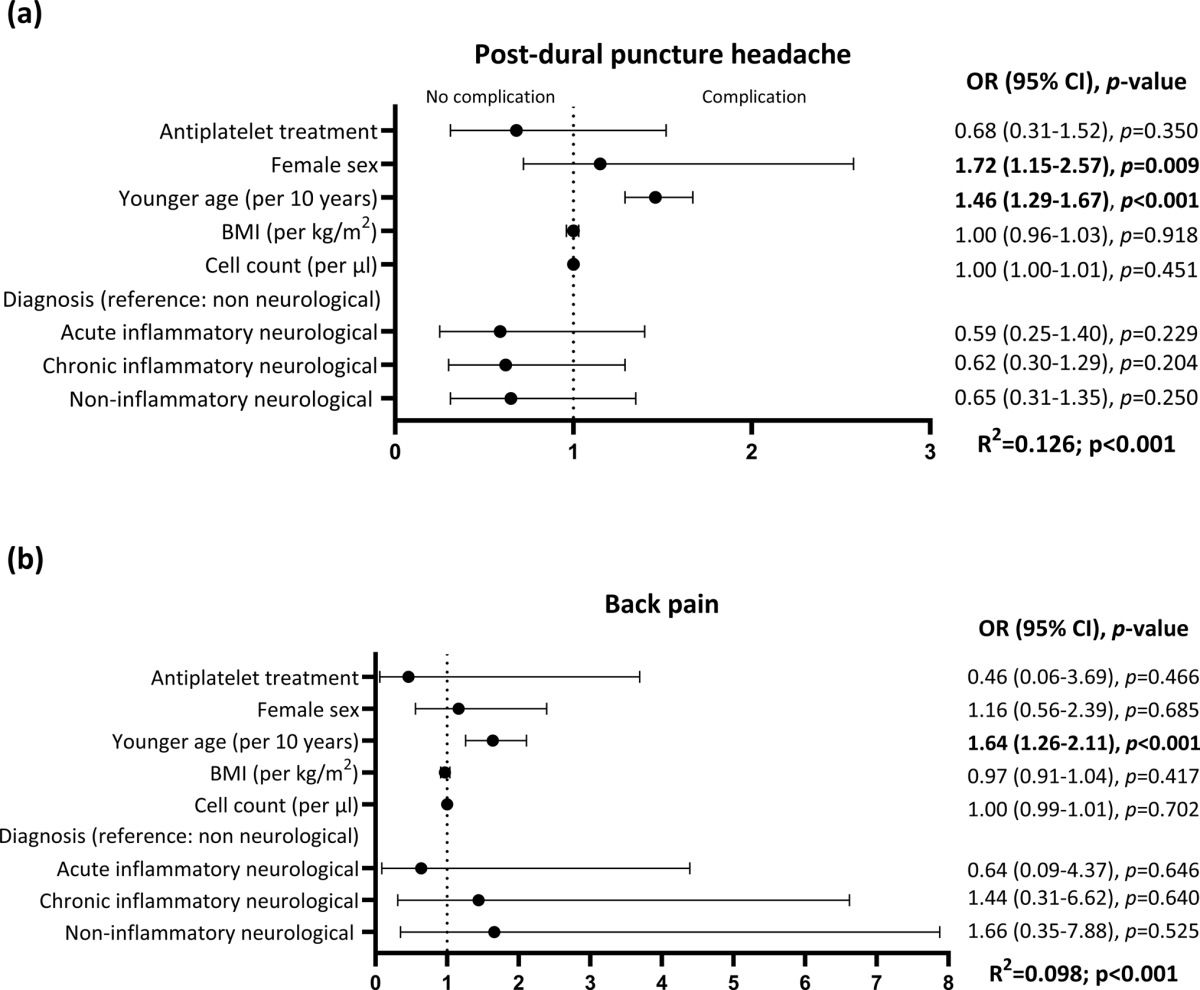
Multivariate logistic regression models for the occurrence of post-dural puncture headache (PDPH) (**a**) and back pain (**b**). *BMI* body mass index

PDPH occurred in 9 (8.1%) and 143 (21.3%) patients with and without APT, respectively (*p* = 0.001). On the other hand, back pain was not associated with APT therapy (2 [1.8%] vs. 40 [6.0%], *p* = 0.072). Univariately, APT (OR 0.33; 95% CI 0.16–0.66; *p* = 0.002) and non-inflammatory neurological diagnosis (OR 0.34; 0.18–0.64; *p* < 0.001) were associated with lower likelihood of PDPH, whereas female sex (OR 1.97; 95% CI 1.36–2.86; *p* < 0.001) and younger age (OR 1.50 per 10 years; 1.33–1.67; *p* < 0.001) were associated with higher likelihood of PDPH. Similarly, younger age (OR 1.64 per 10 years; 95% CI 1.32–2.04; *p* < 0.001) was associated with higher likelihood of back pain (Supplemental Table 4). In the multivariate binary logistic regression model only younger age (OR 1.46 per 10 years; 95% CI 1.29–1.67; *p* < 0.001) and female sex (OR 1.72; 95% CI 1.15–2.57; *p* = 0.009) were associated with higher likelihood of PDPH, whereas APT and other covariates (BMI, cell count and final diagnosis) were not. Similarly, younger age (OR 1.64 per 10 years; 95% CI 1.26–2.11; *p* < 0.001) but not female sex was associated with back pain after LP (Fig. 3).

Prespecified sensitivity analyses excluding patients with other APT than acetylsalicylic acid and dual APT did not significantly alter the overall results or impact individual variables.

## Discussion

The aim of this study was to investigate the incidence of complications after LP and their risk factors in a real-world cohort with a particular focus on the role of APT. Two key findings emerge from this study: (1) complications occur in approximately 23.2% of patients, (2) younger age and female sex are independently associated with higher likelihood of complications, whereas APT is not.

Although LP is a procedure countlessly performed in clinical routine, minor and major complications can occur even following state-of-the-art procedure. In our study, complications occurred in approximately one fourth of patients, which is slightly lower than previously reported [7]. However, the incidence of PDPH varies widely in the literature, partly due to different definitions of PDPH, with most of previous studies using a stricter definition of PDPH in terms of improvement in supine position and associated symptoms (nausea, photophobia, hyperacusis, tinnitus and/or neck stiffness) [28], whereas in our study, PDPH was defined as any headache occurring within 5 days after LP [25]. Moreover, these studies included demographically diverse populations, providing an additional source of high variability in the prevalence of PDPH [5–7]. Unfortunately, the lack of data on the use of traumatic needles, a known factor contributing to a higher complication rate [5–7], did not allow further analysis yet may have contributed to the lower incidence rate observed in our study. On the other hand, the rate of serious complications (one patient with venous sinus thrombosis) was very low and in line with the literature [18, 29].

According to current guidelines, patients with a low thrombotic risk on dual APT should discontinue clopidogrel while continuing acetylsalicylic acid for 1 week prior to elective LP, whereas monotherapy with acetylsalicylic acid does not need to be discontinued [30]. Consistent with this, we found no association between complication rate and APT. In our study, the limited number of patients receiving dual APT, along with insufficient documentation on whether clopidogrel was discontinued, precluded us from drawing any definitive conclusions regarding LP outcomes in this patient population. However, it appears that LP in patients on APT is safe in both acute and elective settings [22–24], although there are some rare cases of subarachnoid hemorrhage and subdural hematoma previously reported [23, 31].

We were also able to confirm the known risk factors for complications after LP, i.e., younger age and female sex [5–7]. Although the factors contributing to the higher prevalence of complications in women are not known, it may be due to a greater sensitivity to pain [32], psychosocial factors (different reporting rates due to socially learned gender roles) [33], and hormonal causes, with estrogen causing a dilation of the cerebral pial vessels [34], possibly triggering a headache, a mechanism similar to that seen in migraine. In a large multicenter study of patients with dementia, older age (≥ 65 years) was associated with a lower likelihood of PDPH without female predominance, suggesting that the sex dependency disappears with increasing age [7]. The decreasing incidence of complications with higher age may be related to lower pain sensitivity [35], and lower CSF pressure in the elderly [36, 37], potentially leading to a more significant leak of CSF through the puncture hole in young adults. In an older study, lower BMI was found to be a risk factor for complications [6], which could not be verified in other studies, including ours [5, 7, 38–41].

Some limitations of this study need to be acknowledged. We were only able to explain 15% of the variance with our model, which means that there may be several other factors contributing to the complication rate. First, the retrospective study design introduced a number of potential biases, although these were partially mitigated by the detailed characterization of patients. Second, due to a lack of standardized documentation, procedural parameters such as needle used, number of puncture attempts, physician experience and patient positioning could not be included in the analysis. Third, due to inadequate documentation, it was not feasible to ascertain whether and for how long APT had been discontinued prior to the LP. Furthermore, the study is limited by a potential selection bias, as most patients included underwent LP in an elective setting since participation in the local CSF database is rarely considered in an acute setting, compounded by the lack of centralized data on all lumbar punctures performed hospital wide. Finally, the prevalence of complications in our study may have been underestimated due to reporting bias, especially in patients with mild symptoms. Still, Rosenbaum sensitivity tests with Hodges-Lehmann Gamma indicated robustness to bias by unidentified confounders.

## Conclusions

In conclusion, complications after LP occur in approximately one fourth of patients, with younger age and female sex being the main risk factors. As APT is not associated with higher likelihood of complications, LP can be performed safely in patients receiving APT. Further studies are needed to confirm our data, especially in patients on dual APT.

## Supplementary Information

Below is the link to the electronic supplementary material.

Supplementary file1 (DOCX 17 KB)

Supplementary file2 (DOCX 20 KB)

Supplementary file3 (DOCX 17 KB)

Supplementary file4 (DOCX 18 KB)

## Data Availability

Data supporting the findings of this study are available from the corresponding author upon reasonable request by a qualified researcher and upon approval by the data-clearing committee of the Medical University Vienna.
