# Supplementary material for: Antiplatelet therapy is not associated with increased risk of complications after lumbar puncture
Source: J Neurol. 2024 Dec 24;272(1):88. doi: 10.1007/s00415-024-12864-6 (PMC11668846; doi:10.1007/s00415-024-12864-6)
Supplement: Supplementary file 1 — Supplementary file1 (DOCX 17 KB) [file 415_2024_12864_MOESM1_ESM.docx]

**Supplemental Table 1.** Collected parameters. ASS: acetylsalicylic acid, CSF: cerebrospinal fluid, LP: lumbar puncture.

| **Demographics and patient-related parameters** | **CSF parameters** |
| --- | --- |
| - Sex - Age - Body mass index - Indication of LP (acute/elective) - Antiplatelet therapy (ASS, Clopidogrel, dual APT) | - CSF cell-count - CSF erythrocyte count - CSF glucose - Glucose ratio (CSF/serum) - CSF total protein - CSF albumin - Albumin quotient (CSF/serum) - CSF IgG - IgG intrathecal production - IgG relative intrathecal fraction - CSF IgA - IgA intrathecal production - IgA relative intrathecal fraction - CSF IgM - IgM intrathecal production - IgM relative intrathecal fraction - Oligoclonal bands in CSF - CSF opening pressure |
| **Procedural parameters** | **Final diagnosis** |
| - Positioning of the patient - Needle type - Diameter of the needle - Length of the needle - Number of attempts - Experience of the physician | - Acute inflammatory neurological diagnosis (e.g. encephalitis, acute isolated optic neuritis, neuroborreliosis) - Chronic inflammatory neurological diagnosis (e.g., multiple sclerosis, chronic inflammatory demyelinating polyneuropathy, CNS vasculitis) - Non-inflammatory neurological diagnosis (e.g., motor neuron diseases, dementia, Parkinson’s disease)   - Idiopathic intracranial hypertension - Non-neurological diagnosis (e.g., dissociative disorders, non-specific white matter lesions, depression) |
