# Supplementary material for: Antiplatelet therapy is not associated with increased risk of complications after lumbar puncture
Source: J Neurol. 2024 Dec 24;272(1):88. doi: 10.1007/s00415-024-12864-6 (PMC11668846; doi:10.1007/s00415-024-12864-6)
Supplement: Supplementary file 2 — Supplementary file2 (DOCX 20 KB) [file 415_2024_12864_MOESM2_ESM.docx]

**Supplemental Table 2**. Demographics of patients receiving APT.

|  | **APT (n=111)** | **No APT (n=672)** | **p-value** |
| --- | --- | --- | --- |
| Female^a^ | 56 (50.5) | 367 (54.6) | 0.415 |
| Age (years)^b^ | 68 (58–74) | 44 (31-60) | **<0.001** |
| BMI (kg/m^2^)^b^ | 25.6 (22.7–29.2) | 24.6 (21.7-27.9) | 0.072 |
| Complications^a^ | 11 (9.9) | 171 (25.4) | **<0.001** |
| PDPH^a^ | 9 (8.1) | 143 (21.3) | **0.001** |
| Back pain^a^ | 2 (1.8) | 40 (6.0) | 0.072 |
| Venous sinus thrombosis^a^ | 0 (0.0) | 1 (0.1) | 0.684 |
| **Diagnosis** |  |  |  |
| Neurological^a^ | 105 (94.6) | 622 (92.6) | 0.441 |
| Acute inflammatory^a^ | 9 (8.1) | 92 (13.7) | 0.104 |
| Chronic inflammatory^a^ | 13 (11.7) | 232 (34.5) | **<0.001** |
| Non-inflammatory^a^ | 83 (74.8) | 298 (44.3) | **<0.001** |
| IIH^a^ | 1 (0.9) | 25 (3.7) | 0.125 |
| Non-neurological^a^ | 6 (5.4) | 50 (7.4) | 0.441 |
| **Setting** |  |  |  |
| Acute^a^ | 0 (0.0) | 11 (1.6) | 0.175 |
| Elective^a^ | 111 (100.0) | 661 (98.4) | 0.175 |
| **Modality** |  |  |  |
| Bedside^a^ | 111 (100.0) | 669 (99.6) | 0.481 |
| CT-guided^a^ | 0 (0.0) | 3 (0.4) | 0.481 |
| **Comorbidities** |  |  |  |
| Cardiovascular^a^ | 65 (58.6) | 117 (17.4) | **<0.001** |
| Metabolic^a^ | 41 (36.9) | 64 (9.5) | **<0.001** |
| Renal^a^ | 4 (3.6) | 20 (3.0) | 0.726 |
| Gastrointestinal^a^ | 11 (9.9) | 38 (5.7) | 0.088 |
| Hematological^a^ | 2 (1.8) | 13 (1.9) | 0.922 |

## ^a^Number (%), ^b^Median (IQR)
