# Supplementary material for: Antiplatelet therapy is not associated with increased risk of complications after lumbar puncture
Source: J Neurol. 2024 Dec 24;272(1):88. doi: 10.1007/s00415-024-12864-6 (PMC11668846; doi:10.1007/s00415-024-12864-6)
Supplement: Supplementary file 3 — Supplementary file3 (DOCX 17 KB) [file 415_2024_12864_MOESM3_ESM.docx]

**Supplemental Table 3.** Univariate analysis for the primary outcome.

|  | **Any complication** | | |
| --- | --- | --- | --- |
|  | **OR** | **95% CI** | **p-value** |
| APT | 0.32 | 0.17- 0.62 | **<0.001** |
| Female sex | 1.96 | 1.39 - 2.78 | **<0.001** |
| Indication (reference: acute) | 0.52 | 0.15 - 1.81 | 0.308 |
| Cell count (per µl) | 1.00 | 1.00 - 1.01 | 0.154 |
| Younger age (per 10 years) | 1.52 | 1.36-1.69 | **<0.001** |
| BMI (per kg/m^2^) | 0.98 | 0.95 - 1.01 | 0.220 |
| Diagnosis (reference: non-neurological) |  | | |
| Acute inflammatory neurological | 0.61 | 0.30 - 1.25 | 0.173 |
| Chronic inflammatory neurological | 0.86 | 0.46 - 1.59 | 0.629 |
| Non-inflammatory neurological | 0.39 | 0.21 - 0.73 | **0.003** |
