# Supplementary material for: Antiplatelet therapy is not associated with increased risk of complications after lumbar puncture
Source: J Neurol. 2024 Dec 24;272(1):88. doi: 10.1007/s00415-024-12864-6 (PMC11668846; doi:10.1007/s00415-024-12864-6)
Supplement: Supplementary file 4 — Supplementary file4 (DOCX 18 KB) [file 415_2024_12864_MOESM4_ESM.docx]

**Supplemental Table 4.** Univariate analysis for the secondary outcomes.

|  | **PDPH** | | | **Backpain** | | |
| --- | --- | --- | --- | --- | --- | --- |
|  | **OR** | **95% CI** | **p-value** | **OR** | **95% CI** | **p-value** |
| APT | 0.33 | 0.16 - 0.66 | **0.002** | 0.29 | 0.07 - 1.22 | 0.091 |
| Female sex | 1.97 | 1.36 - 2.86 | **<0.001** | 1.41 | 0.74 - 2.67 | 0.294 |
| Indication (reference: acute) | 0.64 | 0.17 - 2.43 | 0.510 | 0.56 | 0.07 - 4.49 | 0.586 |
| Cell count (per µl) | 1.00 | 1.00 - 1.01 | 0.204 | 1.00 | 1.00 - 1.01 | 0.387 |
| Younger Age (per 10 years) | 1.50 | 1.33 - 1.67 | **<0.001** | 1.64 | 1.32 - 2.04 | **<0.001** |
| BMI (per kg/m^2^) | 0.98 | 0.95 - 1.02 | 0.397 | 0.96 | 0.90 - 1.03 | 0.296 |
| Diagnosis (reference: non-neurological) |  | | |  | | |
| Acute inflammatory neurological | 0.55 | 0.27 - 1.16 | 0.117 | 0.83 | 0.13 - 5.10 | 0.837 |
| Chronic inflammatory neurological | 0.69 | 0.36 - 1.29 | 0.240 | 2.53 | 0.58 - 11.13 | 0.219 |
| Non-inflammatory neurological | 0.34 | 0.18 - 0.64 | **<0.001** | 1.18 | 0.27 - 5.29 | 0.825 |
